# Feasibility of virtual non-iodine coronary calcium scoring on dual source photon-counting coronary CT angiography: a dynamic phantom study

**DOI:** 10.1007/s00330-024-10806-4

**Published:** 2024-05-24

**Authors:** Magdalena M. Dobrolinska, Lennart R. Koetzier, Marcel J. W. Greuter, Rozemarijn Vliegenthart, Judith van der Bie, Niek H. J. Prakken, Riemer H. J. A. Slart, Tim Leiner, Ricardo P. J. Budde, Domenico Mastrodicasa, Ronald Booij, Dominik Fleischmann, Martin J. Willemink, Marcel van Straten, Niels R. van der Werf

**Affiliations:** 1 Department of Radiology and Nuclear Medicine Rotterdam, Erasmus MC University Medical Center, Rotterdam, The Netherlands; 2 Department of Nuclear Medicine and Molecular Imaging, University of Groningen, University Medical Center Groningen, Medical Imaging Center, Groningen, The Netherlands; 3 Department of Radiology Stanford, Stanford University School of Medicine, Stanford, CA USA; 4 Department of Radiology, University of Groningen, University Medical Center Groningen, Medical Imaging Center, Groningen, The Netherlands; 5 Department of Radiology Rochester, Mayo Clinic, Rochester, MN USA

**Keywords:** X-ray computed tomography, Calcium, Coronary vessels, Imaging phantoms

## Abstract

**Background:**

The aim of our current systematic dynamic phantom study was first, to optimize reconstruction parameters of coronary CTA (CCTA) acquired on photon counting CT (PCCT) for coronary artery calcium (CAC) scoring, and second, to assess the feasibility of calculating CAC scores from CCTA, in comparison to reference calcium scoring CT (CSCT) scans.

**Methods:**

In this phantom study, an artificial coronary artery was translated at velocities corresponding to 0, < 60, and 60–75 beats per minute (bpm) within an anthropomorphic phantom. The density of calcifications was 100 (very low), 200 (low), 400 (medium), and 800 (high) mgHA/cm^3^, respectively. CCTA was reconstructed with the following parameters: virtual non-iodine (VNI), with and without iterative reconstruction (QIR level 2, QIR off, respectively); kernels Qr36 and Qr44f; slice thickness/increment 3.0/1.5 mm and 0.4/0.2 mm. The agreement in risk group classification between CAC_CCTA_ and CAC_CSCT_ scoring was measured using Cohen weighted linear κ with 95% CI.

**Results:**

For CCTA reconstructed with 0.4 mm slice thickness, calcium detectability was perfect (100%). At < 60 bpm, CAC_CCTA_ of low, and medium density calcification was underestimated by 53%, and 15%, respectively. However, CAC_CCTA_ was not significantly different from CAC_CSCT_ of very low, and high-density calcifications. The best risk agreement was achieved when CCTA was reconstructed with QIR off, Qr44f, and 0.4 mm slice thickness (κ = 0.762, 95% CI 0.671–0.853).

**Conclusion:**

In this dynamic phantom study, the detection of calcifications with different densities was excellent with CCTA on PCCT using thin-slice VNI reconstruction. Agatston scores were underestimated compared to CSCT but agreement in risk classification was substantial.

**Clinical relevance statement:**

Photon counting CT may enable the implementation of coronary artery calcium scoring from coronary CTA in daily clinical practice.

**Key Points:**

- *Photon-counting CTA allows for excellent detectability of low-density calcifications at all heart rates*.
- *Coronary artery calcium scoring from coronary CTA acquired on photon counting CT is feasible, although improvement is needed*.
- *Adoption of the standard acquisition and reconstruction protocol for calcium scoring is needed for improved quantification of coronary artery calcium to fully employ the potential of photon counting CT*.

## Introduction

Coronary artery calcium (CAC) is a strong prognostic factor mostly in asymptomatic individuals, however, it also improves risk assessment in symptomatic patients, as is highlighted in American and European guidelines [1, 2]. Increased cardiovascular risk is especially pronounced in patients with > 1000 Agatston scores [3]. For now, calcium scoring CT (CSCT), which enables the calculation of the Agatston score, is an indispensable part of the CCTA scanning protocol. However, with the introduction of spectral CT systems, calcium scoring from CCTA may become feasible.

To enable CAC scoring from CCTA exams, iodine and calcium should be separated. Spectral CT systems now enable material decomposition to distinguish tissues of different average atomic numbers. Approaches for spectral CT include, among others, dual source, dual layer, and photon counting CT (PCCT). PCCT is a new CT technology that allows for counting the number of incoming photons as well as measurement and discrimination of photon energy [4]. It facilitates the discrimination between two substances in more detail, especially materials of higher atomic numbers [5]. So far, only virtual non-contrast (VNC) reconstructions have been available, which can distinguish two substances: soft tissue and iodine [6]. However, as both iodine and calcium are of a relatively high atomic number, this approach was not optimal for efficient material differentiation [7]. A promising new approach is the reconstruction of virtual non-iodine (VNI) images, which enables differentiation between iodine and calcium, and can remove iodine from CCTA images with the preservation of calcifications [8, 9]. As shown by Emrich et al, CAC scoring from CCTA is feasible, however, CAC scores are strongly underestimated [9]. Fink et al investigated the influence of quantum Iterative Reconstruction (QIR) and different virtual mono-energetic levels on CAC scoring from CCTA, but did not find consistent results [10]. Nevertheless, a systematic assessment and optimization of CCTA reconstruction parameters for CAC scoring from CCTA have not been performed.

Therefore, the aim of our current systematic dynamic phantom study was first, to optimize reconstruction parameters of CCTA acquired on PCCT for CAC scoring, and second, to assess the feasibility of CAC scores calculated from these scans, in comparison to reference CSCT.

## Methods

### Phantom

**Fig. 1:**
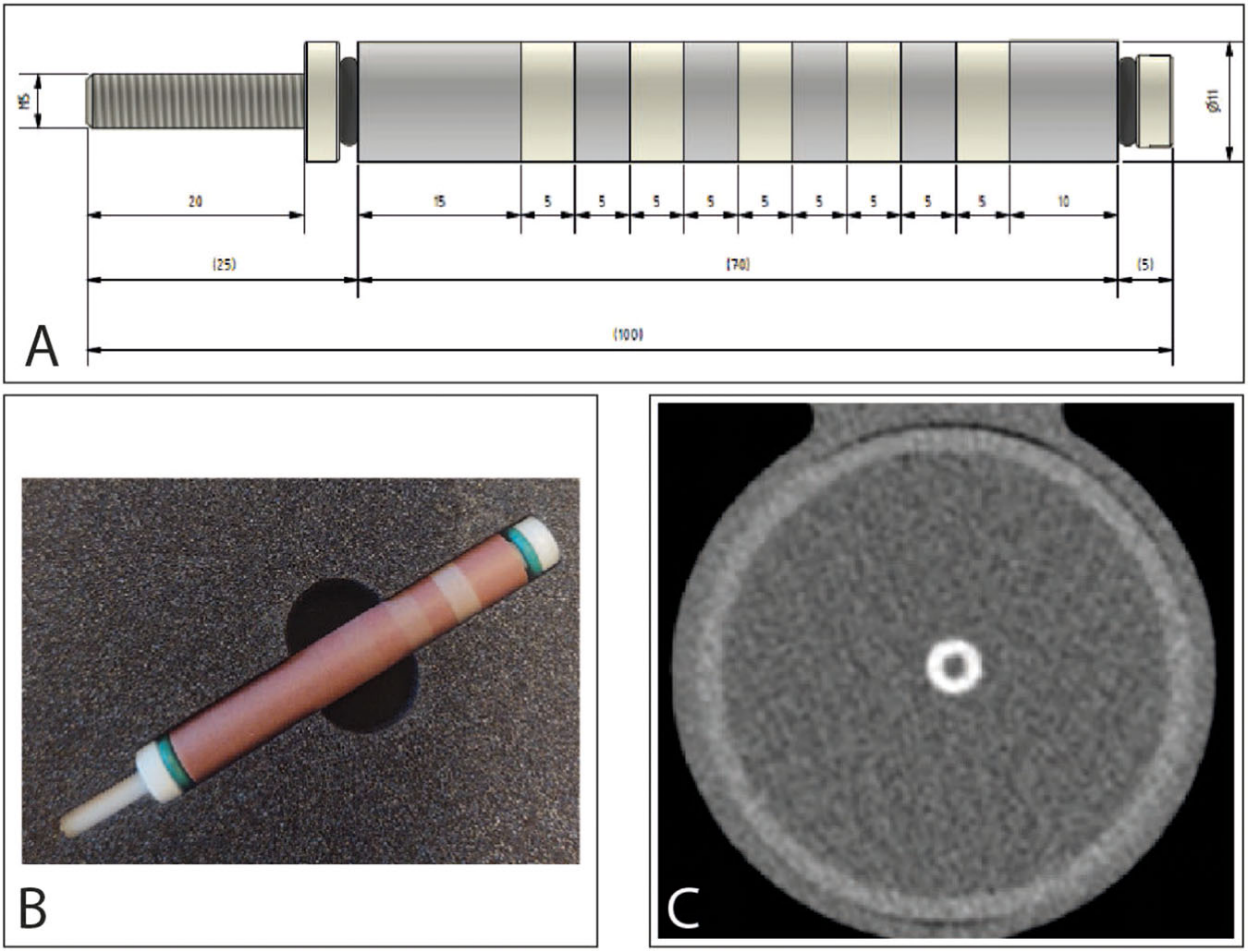
Representations of the hollow artificial artery: (**A**) schematic overview with dimensions in millimeters, with the solid water (grey) and five hydroxyapatite calcifications (yellow) indicated (the fifth calcification’s density was below the calcium threshold, therefore was not included in the analysis), (**B**) Photograph, and (**C**) a 70 keV reconstructed cross-sectional image of the medium density calcification (window width/window level at 750/90 Hounsfield units)

A hollow artificial artery (inner diameter 5 mm, outer diameter 11 mm) was positioned within a water-filled compartment at the center of an anthropomorphic thorax phantom (QRM-thorax, PTW) (Fig. 1). To simulate large patient size, a large fat-tissue equivalent extension ring (Extension Ring Fat L, PTW) was positioned around the thorax phantom, increasing the outer dimensions to 400 × 300 mm [11]. The artery was made of a solid substance with the density of water, with hollow cylindrical hydroxyapatite (HA) calcifications of identical dimensions (inner diameter 5 mm, outer diameter 11 mm, length 5 mm, physical volume 377 mm^3^) but different densities (100, 200, 400, and 800 mg HA/cc, designated as very low, low, medium, and high density, respectively) (Fig. 1). For CSCT scans the artery lumen was filled with water and glucose to mimic in-vivo density of blood (approximately 40 HU at 70 keV). For CCTA scans, the lumen was additionally filled with diluted iodinated contrast agent (Iomeron 350), resulting in approximately 350 HU at a virtual monoenergetic image (VMI) level of 70 keV corresponding to conventional CT images at a tube voltage of 120 kVp. The artery was oriented parallel to the *z*-axis of the CT system. A robotic arm (Sim2D, PTW) translated the artery in the horizontal plane perpendicular to the *z*-axis, at velocities of 0, 10, and 20 mm/s, approximately equivalent to the mean in-vivo velocity of coronary arteries during the scan phase at heart rates of 0, < 60, and 60–75 beats per minute (bpm), respectively [12]. The electrocardiogram output of the robotic arm was coupled to the ECG input of the CT scanner to ensure data acquisition during linear motion of the robot, so without any turning points. To simulate inter-scan variability, each acquisition was repeated five times, with manual repositioning of the setup between each scan (approximately 2 mm translation, and 2 degrees rotation).

### Acquisition and reconstruction parameters

#### CSCT scan

First, the dynamic phantom without iodinated contrast in the lumen was scanned on a dual source PCCT (NAEOTOM Alpha, Siemens Healthineers, Software version Syngo CT VA50) with a clinical CSCT reference protocol: tube potential 120 kVp; axial scan technique; 144 × 0.4 mm collimation; image quality level 16 (Care keV IQ), QIR off, VMI level 70 keV; kernel Qr36; field-of-view 220 mm; matrix 512 × 512; slice thickness/increment 3.0/1.5 mm. Due to motion artefacts depicted on CSCT scans, three scans and the corresponding CCTA scans were excluded from further analysis.

#### CCTA scan

**Fig. 2:**
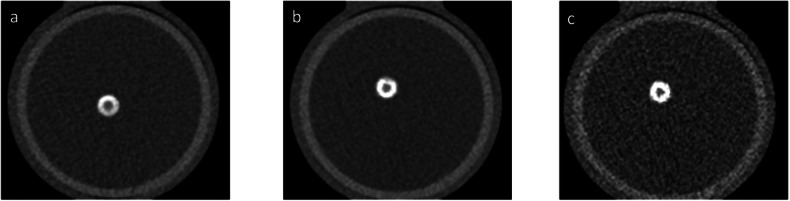
Depiction of non-enhanced CT scan and virtual non-iodine (VNI) reconstructions of the artery: (**a**) non-enhanced CAC scan – the reference calcium scoring CT scan; (**b**) VNI CCTA scan reconstructed with FBP, kernel Qr36f, and 3 mm slice thickness; (**c**) VNI CCTA scan reconstructed with FBP, kernel Qr36f, and 0.4 mm slice thickness. FBP, filtered back projection

Second, the dynamic phantom with iodinated contrast in the lumen was scanned on the same PCCT system with CCTA protocol: tube potential 120 kVp; axial scan technique; 144 × 0.4 mm collimation; image quality level 65 (Care keV IQ, Siemens Healthineers); Quantum Iterative Reconstruction (QIR) off and on (QIR level 2); kernels Qr36 and Qr44f; field-of-view 220 mm; matrix 512 × 512; slice thickness/increment 3.0/1.5 mm and 0.4/0.2 mm (Table 1). All scans were reconstructed with virtual non-iodine (VNI, PURE Calcium, Siemens Healthineers) at 70 keV. An example of reconstruction images is depicted in Fig. 2.

**Table 1:** Summary of CSCT and CCTA protocols

Parameter	Reference CSCT	CCTA
Technique	Axial	Axial
Tube voltage [kVp]	120	120
Automatic exposure control	Clinical CARE keV IQ level 16	Clinical CARE keV IQ level 65
CTDI_vol_	3.3	18.3
Collimation [mm]	144 × 0.4	144 × 0.4
Field of View [mm]	220	220
Rotation time [s]	0.25	0.25
Slice thickness/increment [mm]	3.0/1.5	0.4/0.2 3.0/1.5
Reconstruction kernel	Qr36	Qr44/Qr36
Matrix size [pixels]	Automatic 512 × 512	Automatic 512 × 512
Reconstruction method	QIR off	QIR off, QIR strength 2
monoE level [keV]	70	70
Repetitions	5	5
Artery velocity [mm/s]	0, 10, 20	0, 10, 20
Scan length [cm]	10	10

*CSCT* calcium scoring computed tomography, *CCTA* coronary computed tomography angiography, *IQ* image quality, *QIR* quantum iterative reconstruction, *CTDI*_*vol*_ Volumetric CT dose index

#### Calcium scoring

Agatston and volume scores were automatically determined with a previously validated automated scoring tool, using CT vendor-specific CAC scoring parameters [13]. The default setting for calcium was defined as at least 1 pixel at a threshold of 130 HU [14]. For the 0.4 mm slice thickness, the scores of each slice were multiplied by 0.4/3 to compensate for the non-standard slice thickness. For simulated cardiovascular risk classification, CAC scores derived from the individual calcifications in CCTA (CAC_CCTA_) and CSCT (CAC_CSCT_) were categorized into five risk groups based on the Agatston score (0; 1–100; 101–400; 401–1000; > 1000) [15]. For each velocity and density, CAC_CSCT_ was used as a reference. For volume scores, the physical volume of the calcifications (377 mm^3^) was used as a reference.

To obtain CAC scores from CCTA acquisitions, we acquired several reconstruction settings: QIR off, Qr36f and 3.0 mm slice thickness as standard CSCT reconstruction, and additional reconstructions at QIR level 2, Qr44f, and 0.4 mm slice thickness.

As a first step, calcium detectability was assessed. A calcification was deemed detectable when a non-zero Agatston score could be obtained. Each scan was verified for noise levels, not to include false positive scores. Next, for each combination of reconstruction settings, CAC_CCTA_ was calculated for each calcification and subsequently categorized to one out of five risk groups. Based on the highest agreement in risk categorization, measured using Cohen weighted linear κ, the preferred reconstruction parameters for CAC_CCTA_ were defined.

### Statistical analysis

Categorical variables were presented as percentages. Continuous variables were presented as means (with standard deviations or 95% confidence intervals (95% CI)) or medians with interquartile range (IQR) for normal and non-normal distributions, respectively. Normality of variables was assessed visually based on histograms and q–q plots. Calcification detectability was presented as percentages. The agreement in risk group classification between CAC_CCTA_ and CAC_CSCT_ scoring was measured using Cohen weighted linear κ with 95% CI. Kappa coefficients were categorized as: > 0.0–0.2: slight agreement, > 0.2–0.4: fair agreement, > 0.4–0.6: moderate agreement, > 0.6–0.8: substantial agreement, and > 0.8–1.0: excellent agreement [16]. Statistical analysis was performed using SPSS version 28 (SPSS, IBM).

## Results

### Detectability analysis

On CSCT scans, all calcifications were detected. For most CCTA reconstructions calcium detectability was perfect (100% calcifications detected). Only for the 3.0 mm slice thickness, merely 6.7% of very low-density calcifications and 60% of low-density calcifications were detected (Table 2).

**Table 2:** Calcium detectability of CSCT and CCTA reconstructed with different parameters for very low, low, medium and high-density calcification

Scan description	Very low	Low	Medium	High
CSCT	100%	100%	100%	100%
CCTA (FBP, Qr36f, 3.0 mm)	6.7%	60%	100%	100%
CCTA (FBP, Qr44f, 0.4 mm)	100%	100%	100%	100%
CCTA (FBP, Qr36f, 0.4 mm)	100%	100%	100%	100%
CCTA (QIR, Qr36f, 0.4 mm)	100%	100%	100%	100%

Between brackets reconstruction method, kernel, and slice thickness

*CSCT* calcium scoring computed tomography (reference), *CCTA* coronary computed tomography angiography, *FBP* filtered back projection, *QIR* quantum iterative reconstruction

### CAC scores

#### CAC-scores CSCT without iodinated contrast

**Fig. 3:**
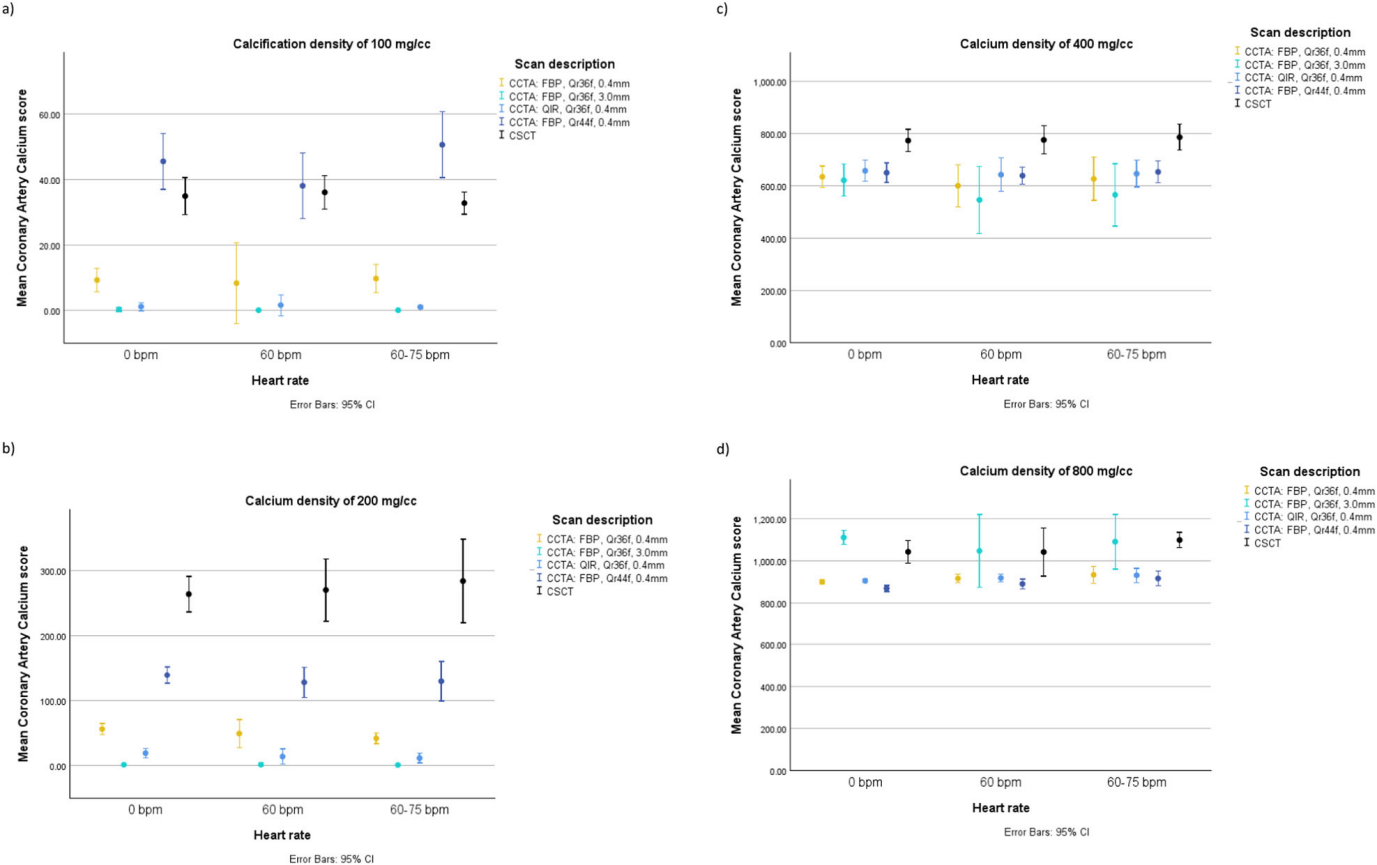
Comparison of Agatston scores from CCTA with different reconstruction parameters and the reference CSCT as a function of heart rate and calcification density. Results presented as mean with 95% CI

At 0 bpm, mean CAC_CSCT_ scores were 35 (95% CI: 31–39), 263 (95% CI: 248–281), 772 (95% CI: 746–798), and 1043 (95% CI: 1007–1073) for very low, low, medium, and high-density CAC, respectively (Fig. 3). The corresponding values at < 60 bpm were 36 (95% CI: 33–39), 269 (95% CI: 245–300), 774 (95% CI: 739–801) and 1042 (95% CI: 971–1093), and at 60–75 bpm they were equal to 33 (95% CI: 31–35), 284 (95% CI: 244–337), 785 (95% CI: 754–821), and 1099 (95% CI: 1071–1123).

#### CAC score - CCTA scans with iodinated contrast

For the very low density calcification, there was no significant difference between CAC_CSCT_ and CAC_CCTA_ reconstructed with QIR off, Qr44f, and 0.4 mm at 0 bpm and < 60 bpm (Fig. 3a). CAC_CCTA_ of the low density calcification differed significantly from CAC_CSCT_ in each reconstruction, showing underestimations of 41%, 53%, and 54% at 0 bpm, < 60 bpm, and 60–75 bpm, respectively (Fig. 3b). For medium density calcifications all CAC_CCTA_ also differed significantly from CAC_CSCT_, with a smallest underestimation of 16%, 18%, and 17% at 0 bpm, < 60 bpm, and 60–75 bpm, respectively (Fig. 3c). For high-density calcifications, there was no significant difference between CAC_CSCT_ and CAC_CCTA_ only for CCTA scans reconstructed with 3.0 mm slice thickness (Fig. 3d).

### Volume score

#### Volume score - CSCT without iodinated contrast

**Fig. 4:**
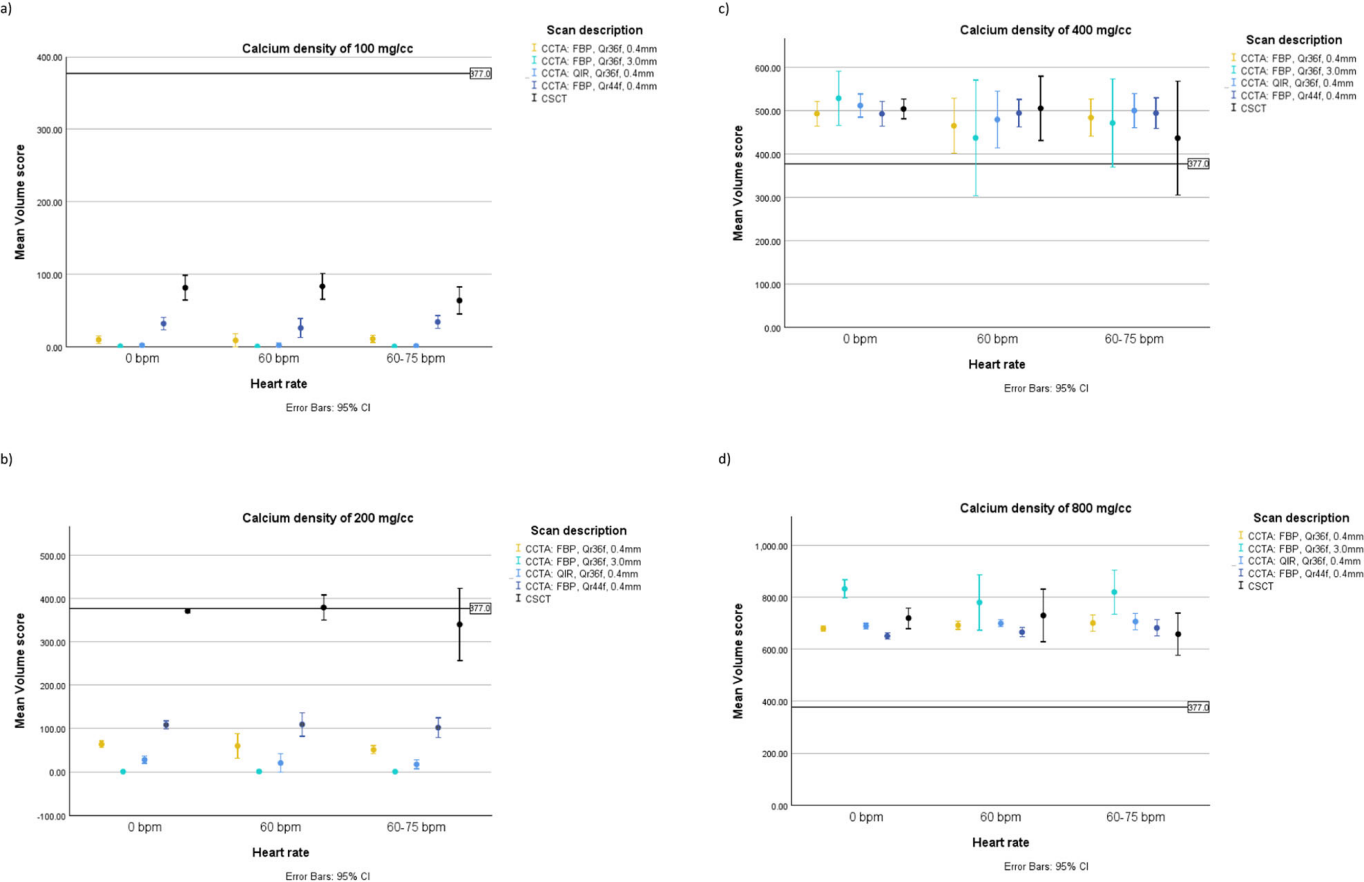
Comparison of Volume scores calculated from CCTA scans with different reconstruction parameters and the reference physical calcification volume (377.0 mm^3^) as a function of heart rate and calcification density. Results presented as mean with 95% CI

The volume scores for very-low and low-density calcifications, obtained from static CSCT scans, were underestimated by 78% and 1.5%, respectively (Fig. 4). Conversely, the volume scores for medium and high-density calcifications, were overestimated by 33% and 94% when calculated from static CSCT, respectively.

#### Volume score - CCTA scans with iodinated contrast

In both static and dynamic acquisitions, for very low, low, and high-density calcifications, the smallest difference between the physical calcification volume and CCTA results was found for the reconstruction with QIR off, Qr44f, and 0.4 mm slice thickness (Fig. 4). At 0 bpm it was underestimated by 92% and 71% for the very low and low-density calcification, respectively (345 ± 7 and 269 ± 7 mm^3^), and overestimated by 72% (273.5 ± 9.6 mm^3^) for high-density calcification. For the medium density calcification, the smallest difference between the physical calcification volume and CCTA results was found for 3.0 mm slice thickness reconstruction with an overestimation of 31%.

At < 60 bpm volume scores were underestimated by 93%, and 73% for very low, and low-density calcification and overestimated by 23%, and 73% for medium and high-density calcification. Similarly, at 60–75 bpm volume scores were underestimated by 91%, and 73% for very low and low density calcification, and overestimated by 24% and 79% for medium and high density calcification.

### Reclassification

The best risk agreement was achieved with QIR off, Qr44f, and 0.4 mm slice thickness, with moderate agreement (κ = 0.762, 95% CI: 0.671–0.853, Table 3a). With QIR off, Qr36f, and 3.0 mm slice thickness reconstruction the risk group agreement was lower, κ = 0.614 (95% CI: 0.524–0.700, Table 3b). The lowest agreement between risk group categorization between CAC_CCCTA_ and CAC_CSCT_ was found with QIR level 2, Qr36f, and 0.4 mm slice thickness reconstruction and QIR off, Qr36f, and 0.4 mm slice thickness reconstruction (κ = 0.590, 95% CI: 0.504–0.676, Table 3c, d). None of the differences in risk agreement described above were statistically significant.

**Table 3:** Agreement in Agatston risk categorization between CSCT and CCTA

a						
		CAC_CSCT_				
CAC_CCTA_	0	1–100	101–400	401–1000	> 1000 AS	Total
0	0	0	0	0	0	0
01–100	0	13	1	0	0	14
101–400	0	0	14	0	0	14
401–1000	0	0	0	15	14	29
> 1000 AS	0	0	0	0	0	0
Total	0	13	15	15	14	57

b						
		CAC_CSCT_				
CAC_CCTA_	0	1–100	101–400	401–1000	> 1000	Total
0	0	12	7	0	0	19
1–100	0	1	8	0	0	9
101–400	0	0	0	1	0	1
401–1000	0	0	0	14	2	16
> 1000	0	0	0	0	12	12
Total	0	13	15	15	14	57

c						
		CAC_CSCT_				
CAC_CCTA_	0	1–100	101–400	401–1000	> 1000	Total
0	0	0	0	0	0	0
1–100	0	13	15	0	0	28
101–400	0	0	0	0	0	0
401–1000	0	0	0	15	14	29
> 1000	0	0	0	0	0	0
Total	0	13	15	15	14	57

d						
		CAC_CSCT_				
CAC_CCTA_	0	1–100	101–400	401–1000	> 1000	Total
0	0	0	0	0	0	0
1–100	0	13	15	0	0	28
101–400	0	0	0	0	0	0
401–1000	0	0	0	15	14	29
> 1000	0	0	0	0	0	0
Total	0	13	15	15	14	57

κ = 0.762, 95% CI: 0.671–0.853

κ = 0.614, 95% CI: 0.524–0.700

κ = 0.590, 95% CI: 0.504–0.676

κ = 0.590, 95% CI: 0.504–0.676

(a) CCTA reconstructed with QIR off, Qr44f, and 0.4 mm; (b) CCTA reconstructed with QIR off, Qr36f, and 3.0 mm; (c) CCTA reconstructed with QIR level 2, Qr36f, and 0.4 mm; (d) CCTA reconstructed with QIR off, Qr36f, and 0.4 mm

## Discussion

Based on this phantom study we can conclude that CCTA virtual non-iodine reconstructions on photon-counting CT with QIR off, Qr44f reconstruction kernel and 0.4 mm slice thickness improve assessment of Agatston score and volume score, as compared to the standard reconstruction. At this reconstruction, the detectability of coronary calcium on virtual non-iodine CCTA scans is perfect for all calcium densities and all heart rates. Nevertheless, Agatston scores of low, medium, and high-density calcifications are underestimated as well as volume scores of very low and low-density calcifications. The Agatston score of very low-density calcification, in turn, is overestimated, as well as the volume score of medium and high-density calcification. In terms of clinical relevance, the simulated risk classification agreement between CCTA and reference CSCT scan is substantial.

With the introduction of virtual non-iodine images, which enables the removal of iodine without affecting the CT numbers of calcium, CAC scoring from contrast-enhanced CCTA is a potentially important opportunity. First steps toward derivation of CAC_CCTA_ were made with dual energy and dual-layer CT systems, with the creation of virtual non-contrast scans. As presented by Schwarz et al, CAC_CCTA_ scores, both Agatston and volume scores acquired on dual–energy CT system and reconstructed with VNC were systematically underestimated [17]. In terms of volume calculation, it was up to 67% lower than the volume derived from CSCT scan [17]. Nevertheless, as this was a clinical study, the physical volume of calcifications remains unknown. Mao et al presented two different algorithms of material decomposition applied on CCTA scans acquired on spectral scans, both of which also underestimated CAC_CCTA_, even up to 85% [18]. Gassert et al used VNC on CCTA scans acquired with a dual-layer CT system and showed CAC_CCTA_ scores up to 75% lower than CAC_CSCT_ [6]. Nadjirji et al went one step further and applied a proportionality factor which improved CAC scoring from CCTA acquired on a dual-layer CT system, however, CAC_CCTA_ was still underestimated by 50% [7]. Based on the abovementioned studies, despite the presented excellent correlation between CAC_CCTA_ and CAC_CSCT_, there was a significant underestimation of CAC_CCTA_ [6, 17, 18]. That might be explained by underestimation of plaque density with the VNC approach, as it only distinguishes between iodine and soft tissue, without calcium discrimination [7, 9]. In our study, we made distinctions among varying calcification densities. The largest percentage underestimation of CAC_CCTA_ occurred with low-density calcifications, reaching a 54% discrepancy at 60–75 bpm. Conversely, for medium and high-density calcifications, the difference was less pronounced, not exceeding 18%.

A crucial element for CAC scoring is CAC detectability, especially of very low-density calcifications. As shown by investigators of ROMICAT II trial, the high-risk plaque, which also includes spotty calcifications, increases the risk of adverse events in patients with stable CAD [16]. In our study, all 5 mm long calcifications of each density, including the very low-density calcification of 100 cc/mg, were detected with 0.4 mm slice thickness. These are significantly better results as compared to a study by Emrich et al, in which 200 cc/mg were not detected on 3 out of 5 repetitions. It might be explained by the fact that Emrich and colleagues used 3 mm slice thickness reconstruction [9]. In our study, when the 3 mm slice thickness reconstruction was used, medium-density calcification (200 cc/mg) remained undetected in 3 out of 15 CCTA scans. Since, all calcifications were detected on the 3 mm CSCT scans, these results indicate that the VNI algorithm needs improvement with respect to detectability on standard 3 mm slices. In any case, it further supports the known need of reconstruction parameter changes to improve calcium detectability.

Our study is the first phantom-based attempt to optimize CCTA reconstruction for CAC scoring. In a previous phantom study, the difference between CAC_CCTA_ reconstructed with VNI and CAC_CSCT_ was about 11% [9]. In that study, however, the phantom did not contain iodine, therefore an investigation of VNI performance in terms of iodine removal, was not possible. In addition, there was no differentiation between investigated calcium densities and the artery phantom was only static [9]. Recently, Mergen et al presented a good agreement between virtual non-contrast scans and true non-contrast scans. However, in this study, CAC was calculated from late enhancement PCCT CCTA, which characterises lower attenuation of coronary arteries as compared to CCTA scans [19]. In our study, the CAC_CCTA_ calculated from VNI was underestimated except for the very low-density calcification. This is in line with previous studies, with the decrease in the difference extent between CAC_CCTA_ and CAC_CSCT_. The abovementioned underestimation of CAC might be explained by suboptimal discrimination of VNI between iodine and calcium. In terms of volume scores, Emrich et al presented 15% underestimation of volume scores derived from CCTA, however, they did not compare this value to the physical volume of calcification, only a comparison to volume scores derived from CSCT was presented [9]. In our study, volume scores derived from CCTA were compared to the known physical calcification volume. Based on our analysis, 0.4 mm slice thickness improves the calculation of volume scores and Agatston scores, which can be explained by the decreased influence of partial volume effects. Importantly, as shown in our analysis, even the volume score derived from CSCT was over- and underestimated, depending on calcium density.

In terms of the reconstruction method, QIR, a standard reconstruction method for CCTA in PCCT, decreased the accuracy of Agatston score and volume score. This finding is also in line with previously presented data by Fink et al, who showed the inconsistency of CAC results derived from QIR reconstructed images [10].

A significant improvement was found when 0.4 slice thickness reconstruction was applied, and the question arises if this score still can be considered an Agatston score and can be compared to the ground truth at 3 mm slice thickness. As presented by Praagh et al, who investigated more robust methods of CAC scoring, thinner slice reconstruction improves inter-scanner reproducibility and increased detectability of low-density calcifications, on CSCT scans acquired with state-of-the-art CT systems [20]. Therefore, to fully employ opportunities of PCCT technology, there is a need to change our methodology of CAC scoring [20–22].

According to clinical guidelines, CAC should be taken into consideration when assessing the risk of cardiovascular events in symptomatic patients [1, 2]. As the crucial point for clinical assessment is calcium detectability, considering an excellent performance of VNI in these terms, on top of substantial risk agreement with reference CSCT scans, CAC scoring from CCTA acquired on PCCT might be feasible.

### Limitations

This phantom study comes with some limitations. The size calcifications were relatively large as compared to calcifications usually visible in vivo. In addition, the artery lumen was relatively large and mostly comparable to the left main artery. However, at this moment it is the only available dynamic phantom that enables plaque morphology assessment on spectral CT. Therefore, to advance the widespread clinical use, the results of this study need further validation in a patient study on smaller coronary arteries. Next, only phantom data was included in the current analysis. In addition, the analysis of reclassification is very limited because only four calcifications were scanned several times. Nevertheless, as this classification might be used clinically in symptomatic patients, we decided to apply this analysis to simulate the clinical information gained. Moreover, we do agree that 0.4 mm slice thickness does not follow the Agatston methodology, nevertheless, due to novel CT scanners and detectors technology, there is a need for a novel calcium scoring method, which is more robust and meets current expectations.

## Conclusions

Based on this dynamic phantom study we conclude that the detection of low-density calcifications is excellent using CCTA acquired from PCCT with VNI. While Agatston scores are generally underestimated compared to CSCT, the agreement in risk classification is substantial. Volume score estimations vary by density, but this pattern also exists in reference CSCT scans. While this suggests CCTA-based CAC assessment on PCCT is feasible, further clinical studies are needed.

## Abbreviations

CAC: Coronary artery calcium
HA: Hydroxyapatite
HU: Hounsfield Units
keV: Kilo electron volt
VMI: Virtual monoenergetic image
VNI: Virtual nono-iodine
PCCT: Photon Counting CT
